# Hepatitis B Virus X Protein Impairs Hepatic Insulin Signaling Through Degradation of IRS1 and Induction of SOCS3

**DOI:** 10.1371/journal.pone.0008649

**Published:** 2010-03-23

**Authors:** KyeongJin Kim, Kook Hwan Kim, JaeHun Cheong

**Affiliations:** Department of Molecular Biology, College of Natural Sciences, Pusan National University, Busan, Republic of Korea; University of Hong Kong, Hong Kong

## Abstract

**Background:**

Hepatitis B virus (HBV) is a major cause of chronic liver diseases, and frequently results in hepatitis, cirrhosis, and ultimately hepatocellular carcinoma. The role of HCV in associations with insulin signaling has been elucidated. However, the pathogenesis of HBV-associated insulin signaling remains to be clearly characterized. Therefore, we have attempted to determine the mechanisms underlying the HBV-associated impairment of insulin signaling.

**Methodology:**

The expressions of insulin signaling components were investigated in HBx-transgenic mice, HBx-constitutive expressing cells, and transiently HBx-transfected cells. Protein and gene expression was examined by Western blot, immunohistochemistry, RT-PCR, and promoter assay. Protein-protein interaction was detected by coimmunoprecipitation.

**Principal Findings:**

HBx induced a reduction in the expression of IRS1, and a potent proteasomal inhibitor blocked the downregulation of IRS1. Additionally, HBx enhanced the expression of SOCS3 and induced IRS1 ubiquitination. Also, C/EBPα and STAT3 were involved in the HBx-induced expression of SOCS3. HBx interfered with insulin signaling activation and recovered the insulin-mediated downregulation of gluconeogenic genes.

**Conclusions/Significance:**

These results provide direct experimental evidences for the contribution of HBx in the impairment of insulin signaling.

## Introduction

An estimated 2 billion people worldwide are currently infected with the hepatitis B virus (HBV), which results in chronic hepatitis, cirrhosis, and in certain instances, hepatocellular carcinoma (HCC) [1], [2]. Among the four proteins originating from the HBV genome, such as the polymerase, surface, core, and HBx proteins, hepatitis B virus X, a small 154-amino acid protein, is a multifunctional regulator which modulates a variety of host processes via interaction with virus and host factors [3], [4]. Previous reports have demonstrated that HBx proteins induce the expression of lipid synthesis-related genes and inflammation in transgenic mice [5], [6], [7]. Generally, hepatic steatosis, the accumulation of lipid in the hepatocytes, has negative effects on liver functions, which may be resulted or caused by inflammation. NF-κB is activated in the hepatocytes and cytokines including interleukin-6 (IL-6), tumor necrosis factor-alpha (TNF-α), and interleukin-1 beta (IL-1β) are overproduced in fatty liver. These proinflammatory cytokines can participate in the attenuation of insulin signaling [8], [9], [10].

Insulin signaling is mediated by a complex, highly integrated network, which controls several processes. In the response of insulin, insulin receptor (IR) phosphorylates insulin receptor substrate (IRS) proteins, which are linked to the activation of two main signaling pathways: the phosphatidylinositol 3-kinase (PI3K)–Akt/protein kinase B (PKB) pathway, which is responsible for the majority of the metabolic actions of insulin, and the Ras–mitogen-activated protein kinase (MAPK) pathway, which regulates the expression of some genes and cooperates with the PI3K pathway to control cell growth and differentiation [11]. In the liver, insulin is involved in a number of actions responsible for glucose control and lipid metabolism. In relations with glucose metabolism in liver, insulin regulates the glucose concentration by inhibiting hepatic glucose production and stimulating glycogen synthesis. On a molecular level, increased hepatic glucose production is regulated by insulin, which can inhibit the expression of key gluconeogenic enzymes, phosphoenolpyruvate carboxykinase (PEPCK) and glucose-6-phosphatase (G6Pase) in normal states. Also, insulin is a strong activator of the lipogenic pathway through activation of lipogenic transcription factors, such as SREBP-1 and ChREBP [12].

Suppressor of cytokine signaling (SOCS) proteins and cytokine-inducible SRC homology 2 (SH2)-domain-containing (CIS) proteins constitute a family of intracellular proteins that play major roles in immune cell proliferation, differentiation, migration, and modulation of immune responses [13], [14]. A mechanism for interleukin-6 signaling in the liver has been previously proposed, which involved the activation of signal transducer and activator of transcription 3 (STAT3) and the subsequent induction of SOCS, a negative regulator of cytokine signaling [15], [16], [17]. Activated STATs are translocated to the nucleus where they bind to STAT response elements (SREs) on target genes to regulate transcription. SOCS3 is a direct target of this signaling cascade, and acts in a negative feedback loop to inhibit STAT phosphorylation at the receptor complex [18]. In the recent report, hepatic SOCS3 is a mediator of insulin resistance in the liver; however, the hepatocyte-specific SOCS3-deficient mice promote systemic insulin resistance by mimicking chronic inflammation [19]. Generally, the induction of SOCS proteins inhibits insulin signaling via several distinct mechanisms, including direct interference with insulin receptor activation, the blockage of IRS activation, and the induction of proteasome-mediated IRS degradation [20], [21].

Although recent evidence suggests that chronic hepatitis C is associated with increased risk of development of insulin resistance [22], the studies linking HBV to insulin resistance or diabetes were less identified. However, some reports demonstrated their relationship. Custro *et al.* examined that incidence of diabetes mellitus in adults with CHB is four time higher than that in the general population [23]. Also, patients with chronic hepatitis have impaired glucose metabolism with hyperinsulinemia and insulin resistance [24]. In another point of view, a report defined that a high frequency of HBV infection is identified in diabetes patients [25]. Based on previous studies, we hypothesize that HBx-induced lipid accumulation and inflammation in the liver can negatively effect on hepatic insulin signaling. In this study, we have characterized the components of the insulin signaling cascades in the context of HBx protein expression. Herein, we demonstrate that the level of IRS1 is concomitantly downregulated in HBx-expressing liver tissues and cells via the persistent induction of SOCS3 proteins. We also examine that STAT3 is activated in HBx protein expression and C/EBPα and STAT3 can increase the SOCS3 expression in the transcriptional level. Furthermore, we present evidence suggesting that HBx interferes with the activation of insulin signaling, thereby resulting in the inhibition of the activities of insulin, including gluconeogenic gene expression. This may clarify the molecular mechanisms by which HBx expression is associated with the hepatic insulin signaling.

## Methods

### Plasmids, reagents, and antibodies

pcDNA3/6myc/SOCS3 were a generous gift from Dr. Akihiko Yoshimura [26]. pGL3B/SOCS3 promoter, pSUPER/GFP control, and pSUPER/SOCS3 were generously provided by Dr. Fred Schaper [27], HBV 1.2-mer wild-type and HBx null mutant vectors derived from the HBV *ayw* subtype were kindly provided by Dr. Wang-Shick Ryu [28], [29]. m67-Luc construct (high-affinity binding site for Stats followed by the luciferase gene) was kindly provided from Dr. Jacqueline Bromberg [30]. pcDNA3/HA/HBx derived from HBV *adr* subtype, pGL3B/PEPCK promoter, and pCMX1/C/EBPα were previously described [6], [31], [32].

The transfection reagents PolyFect and JetPEI were purchased from QIAGEN and Polyplus-transfection, respectively. All other reagents were purchased from Sigma. The antibodies against IRS1 (sc-559), Myc (sc-789), C/EBPα (sc-61), Akt1/2 (sc-8312), and SOCS3 (sc-9023) were purchased from Santa Cruz Biotechnology, INC. and Actin (A2066), HBx (MAB8419), HA (1 867 423), Phospho-Akt (Ser473) (#9271), PI3K-p85 (#4292), Phospho-PI3K-p85 (Tyr458) (#4228), STAT3 (#9132), Phospho-STAT3 (Tyr705) (#9131), Phospho-Tyr (#9411), Phospho-IRS1 (Ser307) (07-247), and Insulin receptor β (07-724) antibody were obtained from Sigma, Chemicon, Roche, Cell Signaling, and Upstate, respectively.

### Cell culture, transient transfection, and stable transfection

HepG2 cell lines were maintained in DMEM-10% fetal bovine serum (Gibco BRL). Transient transfections were conducted using PolyFect or JetPEI reagents of cell cultures in 24-well culture plates with the indicated reporter plasmids, then cotransfected with mammalian expression vectors. Expression vectors were maintained at constant total amounts via the addition of empty vectors. Relative luciferase activities were assessed by luciferin (BD Biosciences). HepG2/HA and HepG2/HA/HBx stable transfectants were as previously described [6], [7], and maintained in DMEM-10% FBS containing 200 µg/ml of G418.

### RNA isolation, reverse transcriptase-polymerase chain reaction, and quantitative real-time PCR

Total RNA from HepG2 cells was prepared using TRIzol (Invitrogen) in accordance with the manufacturer's recommendations. The cDNA was synthesized from 2 µg of total RNA with Moloney murine leukemia virus (MMLV) Reverse Transcriptase (Promega) with a random hexamer (Cosmo, Korea) at 37°C for 1 hour. A 1/25^th^ aliquot of the cDNA was subjected to PCR amplification using gene-specific primers (Table 1). Real-time PCR was performed with an SYBR Green I LightCycler-based real-time PCR assay (Roche Applied Science). The reaction mixtures were prepared using LightCycler Fast Start DNA master mixture for SYBR Green I, 0.5 µM of each primer, 4 mM MgCl_2_. All PCR conditions and primers optimized to produce a single product of the correct base pair size.

**Table 1:** Primers for the RT-PCR amplification.

Gene	Species	Sense	Antisense
IRS1	Human/Murine	GGAGTACATG AAGATGGACC TGG	CTGTTCGCAT GTCAGCATAG C
SOCS1	Human	GAGAGCTTCG ACTGCCTCTT	AGGTAGGAGG TGCGAGTTCA
SOCS3	Human	TCCCCCCAGA AGAGCCTATT AC	TACTGGTCCA GGAACTCCCG
SOCS3	Murine	GCTGGCCAAA GAAATAACCA	AGCTCACCAG CCTCATCTGT
C/EBPα	Human	GAGTGGCGGC AGCGGC	CAGTTCGCGG CTCAGCTGTT
PEPCK	Human	CAGGCGGCTG AAGAAGTATG A	CGTCAGCTCG ATGCCGATCT T
G6Pase	Human	GTGGATTCTC TTTGGACAGC G	CAGACAGACA TTCAGCTGCA CA
HBx	Human/Murine	TTCTCATCTG CCGGTCCGTG	GGGTCAATGT CCATGCCCCA
β-actin	Human/Murine	GACTACCTCA TGAAGATC	GATCCACATC TGCTGGAA
GAPDH	Human	GTGGTCTCCT CTGACTTCAA	TCTCTTCCTC TTGTGCTCTT G
GAPDH	Murine	CCATGTTTGT GATGGGTGTG AACC	TGTGAGGGAG ATGCTCAGTG TTGG

### Immunohistochemistry

The liver tissues of wild-type and HBx-transgenic mice derived from the HBV *adr* subtype genome were kindly provided from Dr. Je Kyung Seong. The production of HBx-transgenic mice has already been reported [6], [7], [33]. The liver tissues were fixed in 4% formaldehyde. In brief, the paraffin-embedded tissue sections were deparaffinized and hydrated with distilled water. Immunohistochemical staining was conducted using a DAB system (Zymed Laboratories INC.) in accordance with the manufacturer's instructions. The anti-IRS1 and SOCS3 antibodies were diluted to 1∶100 with blocking buffer. As a negative control, the primary antibody was replaced with normal immunoglobulin.

### Chromatin immunoprecipitation assay

ChIP assays were conducted as described by the manufacturer (Upstate Biotechnology, Lake Placid, NY) with some modifications. The chromatin solutions were sonicated and incubated with anti-C/EBPα or control IgG, then rotated overnight at 4°C. Chromatin DNA was purified and subjected to PCR analysis. In order to amplify the human SOCS3 promoter regions harboring C/EBPs binding sites, the following primer sets were utilized; sense: 5′-CTC GCG GCC CGC CCT CGG-3′, antisense: 5′-GCT GCG TGC GGG GCC GAA GC-3′. After amplification, the PCR products were resolved on 1.5% agarose gel and visualized via ethidium bromide staining.

### Coimmunoprecipitation

The cells were lysed via the addition of radioimmunoprecipitation assay (RIPA) buffer, then incubated for 10 minutes on ice and then scraped into microcentrifuge tubes. After 15 minutes of centrifugation, an aliquot of the lysates was removed for Western blotting, and the remainder was immunoprecipitated overnight with 1.5 µg anti-C/EBPα, anti-HA, anti-STAT3, and anti-IRS1, and 40 µl of protein G or A-Sepharose (50% suspension). Lysates and immunoprecipitates were then separated via SDS-PAGE and transferred onto PVDF membranes for blotting. The proteins were detected using horseradish peroxidase-conjugated secondary antibodies and visualized via chemiluminescence.

### Statistical analysis

Statistical analyses were conducted via unpaired or paired *t* tests, as appropriate. All data were expressed as the means ± SD. *P* values of <0.05 were considered to be significant.

## Results

### HBx induces degradation of insulin receptor substrate 1 via the ubiquitin-proteasome pathway

**Figure 1:**
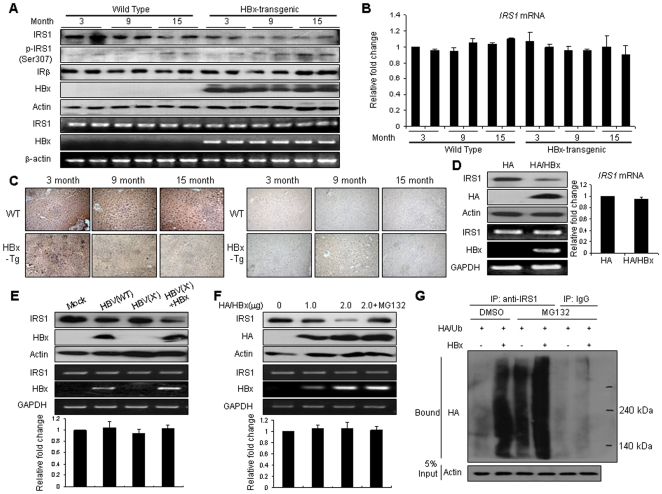
HBx decreases IRS1 expression. (A) The effects of HBx protein on IRS1 expression in wild-type and HBx-transgenic mice. Proteins in liver extracts from control and HBx-transgenic mice were immunoblotted with anti-IRS1, anti-phospho-IRS1 (Ser307), anti-IRβ, anti-HBx, and anti-Actin antibodies. Total RNA was isolated from liver tissues, and the levels of IRS1, HBx, and β-actin mRNA were identified by RT-PCR. (B) The IRS1 mRNA expression in wild-type and HBx-transgenic mice. Total RNA was extracted from liver tissues, and the levels of IRS1 mRNA were determined by quantitative real-time PCR. The data were normalized relative to the GAPDH mRNA. (C) *Left*; Immunohistochemistry of IRS1 in livers of wild-type and HBx-transgenic mice. *Right*; isotype controls of immunohistochemistry for IRS1 antibodies. (D) The effect of HBx protein on IRS1 expression in HepG2/HA/HBx stable cells. HepG2 cell extracts stably transfected with HA-tagged HBx protein were immunoblotted with the IRS1 antibody. Total RNA was also isolated from Mock or HBx stable cells and subjected to RT-PCR analysis (*left*) or quantitative real-time PCR (*right*) with primers for IRS1. (E) The effect of the HBV replicon on IRS1 protein expression. HepG2 cells were transfected with the 1.2-mer replicon (a greater-than-genome-length) construct, a wild-type (WT) or HBx-null mutant (X^−^), and HBx. Total cell extracts were subjected to Western blot analysis with IRS1 antibodies and total RNA was isolated from the replicon-transfected cells, and the levels of IRS1, HBx, and GAPDH mRNA were identified by RT-PCR (*upper*) or quantitative real-time PCR (*bottom*). The data were normalized relative to the GAPDH mRNA. (F) The effect of HBx on IRS1 protein expression in HepG2 cells. HA-tagged HBx protein was transiently expressed for indicated concentrations in HepG2 cells. 48 hours after transfection, MG132 (25 µM) was added for 2 hours. Total RNA was isolated from the cells, and the levels of IRS1, HBx, and GAPDH mRNA were identified by RT-PCR (*upper*) or quantitative real-time PCR (*bottom*). The data were normalized relative to the GAPDH mRNA. (G) The identification of ubiquitinated IRS1 by HBx proteins. HA-tagged ubiquitin was expressed in Mock or HBx stable cells in the treatment of MG132, or not expressed. Whole-cell lysates were immunoprecipitated with IRS1 antibodies and immunoblotted with HA antibodies.

In order to assess the effects of HBx on IRS1 in the liver, we assessed the expression of IRS1 protein using immunoblotting and immunohistochemistry, and determined that IRS1 was significantly downregulated in the liver tissues of 9 and 15-month-old transgenic mice (Figure 1A and 1C). The phosphorylation of Ser307 of IRS1, which is located in the phosphotyrosine binding (PTB) domain, has been correlated with negative regulation of insulin signalling [11], [34]. Its phosphorylation of IRS1 can inhibit insulin-stimulated tyrosine phosphorylation of IRS1 and block interactions with the insulin receptor and inhibit insulin action [35]. We noted that the Ser307 phosphorylation levels of IRS1 proteins were higher in the HBx-transgenic mice than in the controls, even though total IRS1 protein levels were reduced. However, protein expressions of IRβ were not reduced. We detected no differences in the mRNA levels of IRS1 of the transgenic mice in the RT-PCR (Figure 1A) and quantitative real-time PCR (Figure 1B).

As shown in Figure 1D, western blot analysis consistently demonstrated that the levels of IRS1 protein in three independent HBx-stable transfectants were lower than in the parental cell lines. We observed no differences in the mRNA levels of IRS1 in the HBx-stable transfectants in RT-PCR (*left*) and quantitative real-time PCR (*right*). We surmised that the expression level of the HBx induced by a strong promoter in the transfection system is assured to be greater than the physiological level expressed during chronic HBV infection [7], [28], [36]. In consideration of this concern, we assessed the effects of a modest level of HBx expression from a HBV replicon, a greater-than-genome-length construct (1.2-mer). As shown in Figure 1E, the transfection of the wild-type HBV 1.2-mer (WT) in HepG2 cells resulted in the abrogation of IRS1 protein levels, whereas the transfection of the HBx-null mutant (X^−^) of HBV 1.2-mer did not reduce its expression as compared to the wild-type. Furthermore, the ectopically expressed HBx could recover the suppression of the HBx-null mutant (X^−^) of HBV 1.2-mer in IRS1 protein expression. The mRNA levels of IRS1 were unchanged in the replicon-transfected cells. The transient transfection of HBx into HepG2 cells induced a significant suppression of protein levels of IRS1 in a dose-dependent manner. However, a proteasome inhibitor, MG132, recovered the IRS1 protein level in HBx-transfected cells and mRNA levels of IRS1 were not changed (Figure 1F). We subsequently attempted to determine whether IRS1 was ubiquitinated for protein degradation in HBx-transfected cells. HBx promoted the ubiquitination of IRS1, particularly in the treatment of MG132 (Figure 1G). These results support the notion that HBx can downregulate IRS1 proteins in both the liver of transgenic mice and hepatoma cell lines expressing the HBx proteins.

### HBx induces SOCS3 expression

**Figure 2:**
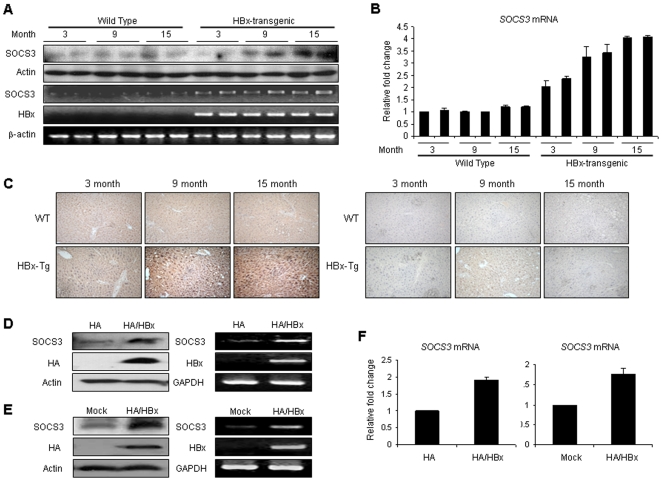
HBx increases SOCS3 expression. (A) The protein and mRNA expression of SOCS3 in wild-type and HBx-transgenic mice in a stage-specific manner. The liver tissues of control mice and HBx homozygous transgenic mice were immunoblotted with anti-SOCS3 antibody. (B) The SOCS3 mRNA expression in wild-type and HBx-transgenic mice. Total RNA was extracted from liver tissues, and the levels of SOCS3 mRNA were determined by quantitative real-time PCR. The data were normalized relative to the GAPDH mRNA. (C) *Left*; Immunohistochemistry of SOCS3 in the livers of wild-type and HBx-transgenic mice. *Right*; isotype controls of immunohistochemistry for SOCS3 antibodies. (D) The effect of HBx protein on SOCS3 protein and mRNA expression in stable cell lines. HepG2 cell extracts stably expressing HA-tagged HBx protein were immunoblotted with the SOCS3 antibody (*left*). Total RNA was also isolated from HBx-constitutive expressing cells and subjected to RT-PCR analysis with primers for SOCS3 (*right*). (E) The protein and mRNA expression of SOCS3 in transiently HBx-transfected cells. HA-tagged HBx were transiently expressed in HepG2 cells. (F) The mRNA expression of SOCS3 in stable cells (*left*) or transiently HBx-transfected cells (*right*). The levels of SOCS3 mRNA were examined by quantitative real-time PCR. The data were normalized relative to the GAPDH mRNA.

Because previous reports have revealed a mechanism by which the action of insulin is inhibited as the result of the increased ubiquitination and degradation of IRS1 via SOCS1 and SOCS3 [20], we first evaluated the expression levels of SOCS1 and SOCS3 proteins in the transgenic mice and HBx-transfected cells. Although levels of SOCS1 protein and mRNA were not induced, SOCS3 protein and mRNA levels were significantly higher in the HBx-transgenic mice, particularly in the 9 and 15-month stages, as compared to the control mice (Figure 2A and 2B). The results of immunohistochemisty demonstrated that the levels of SOCS3 protein were induced in the 9 and 15-month-old transgenic mice (Figure 2C). Furthermore, SOCS3 mRNA levels significantly trended toward an increase in stable cells expressing the HBx proteins, and these results were consistent with the protein level observed in the immunoblotting results (Figure 2D). We then evaluated the induction of SOCS3 mRNA and protein levels in cells transiently transfected with HBx. As shown in Figure 2E, cells transfected with HBx evidenced a statistically significant induction of their mRNA levels as compared to control cells that were transfected with empty vectors. The protein level of SOCS3 was also upregulated in the HBx-expressing cells. The mRNA induction in quantitative real-time PCR was confirmed for SOCS3 expression in stabley or transiently HBx expression cells (Figure 2F). Taken together, these results suggest that the expression of SOCS3 can be regulated by HBx proteins.

### HBx enhances SOCS3-induced IRS1 protein degradation

**Figure 3:**
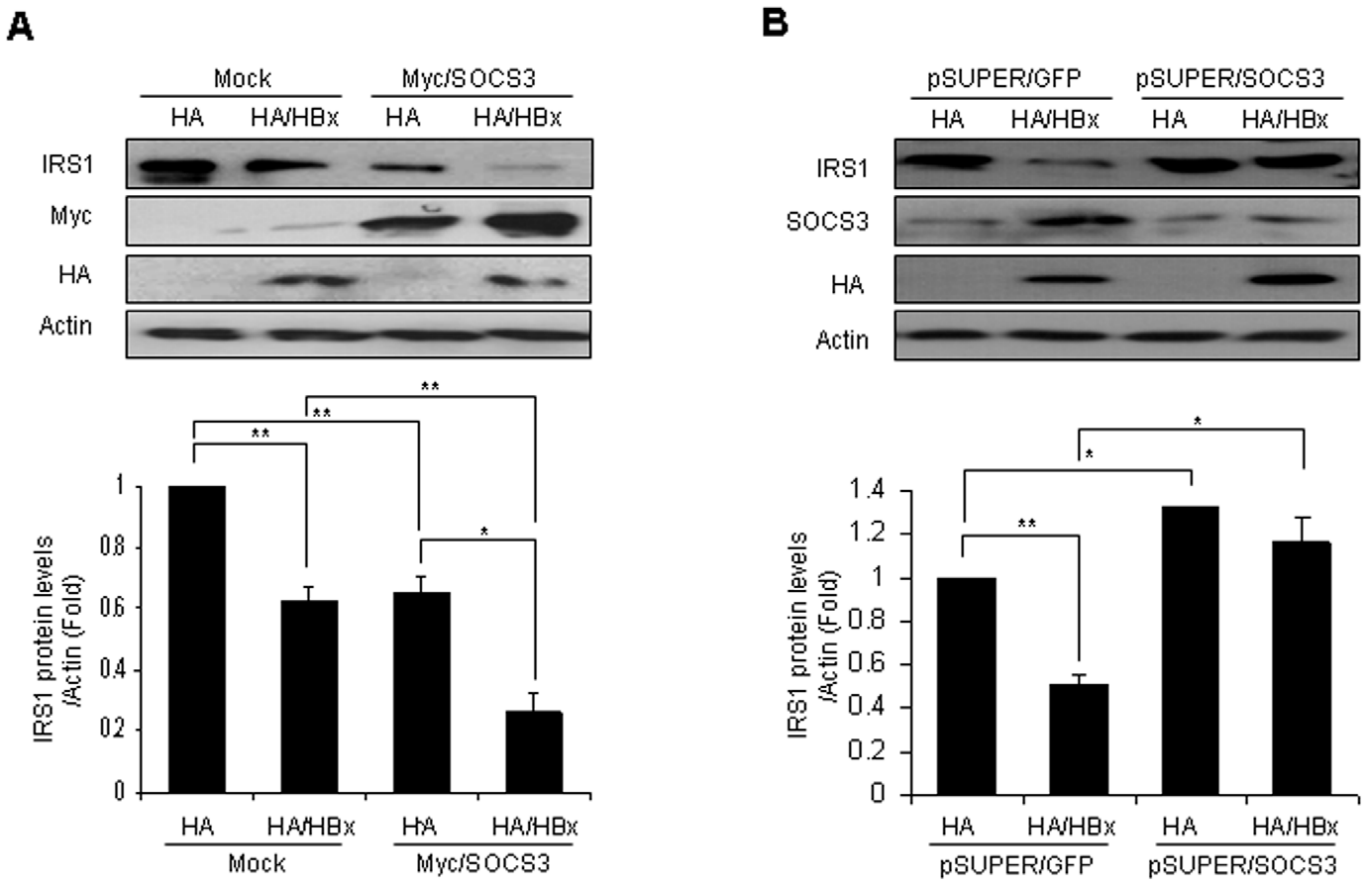
SOCS3 is required for HBx-induced IRS1 protein expression. (A) The degradation of IRS1 by cooperation between HBx and SOCS3. *Top*; Mock and HBx-constitutively expressing HepG2 cells were transiently transfected with Myc-tagged SOCS3. 48 hours after transfection, the cell extracts were immunoblotted with the IRS1-specific antibodies. *Bottom*; Image analysis of IRS1 protein expression by densitometry. (B) The effect of HBx and SOCS3 siRNA on IRS1 protein expression. *Top*; Mock and HBx-constitutive expressing HepG2 cells were transiently transfected with pSUPER/GFP control or pSUPER/SOCS3. The cell extracts were immunoblotted with the IRS1-specific antibodies. *Bottom*; Image analysis of IRS1 protein expression by densitometry. Values were expressed as the means ± SD. *P<0.05 and **P<0.01 compared with indicated controls.

As noted above, the SOCS3 proteins have been previously reported to induce the proteasome-dependent degradation of IRS1, and HBx may result in the induction of SOCS3 mRNA and protein expression. In order to determine whether HBx-induced SOCS3 expressions result in the reduction of IRS1 protein levels, we cotransfected the SOCS3 expression vector into HBx-stable cell lines. As shown in Figure 3A, the further downregulation of IRS1 was observed in the coexpression of SOCS3 proteins. Furthermore, we attempted to determine whether RNAi against SOCS3 could markedly recover IRS1 protein levels (Figure 3B), and the polyubiquitination of IRS1 was barely observed in the coexpression of HBx and RNAi for SOCS3 (data not shown). These data indicate that HBx promotes the SOCS3-mediated downregulation of the IRS1 protein.

### C/EBPα regulates HBx-induced SOCS3 expression

**Figure 4:**
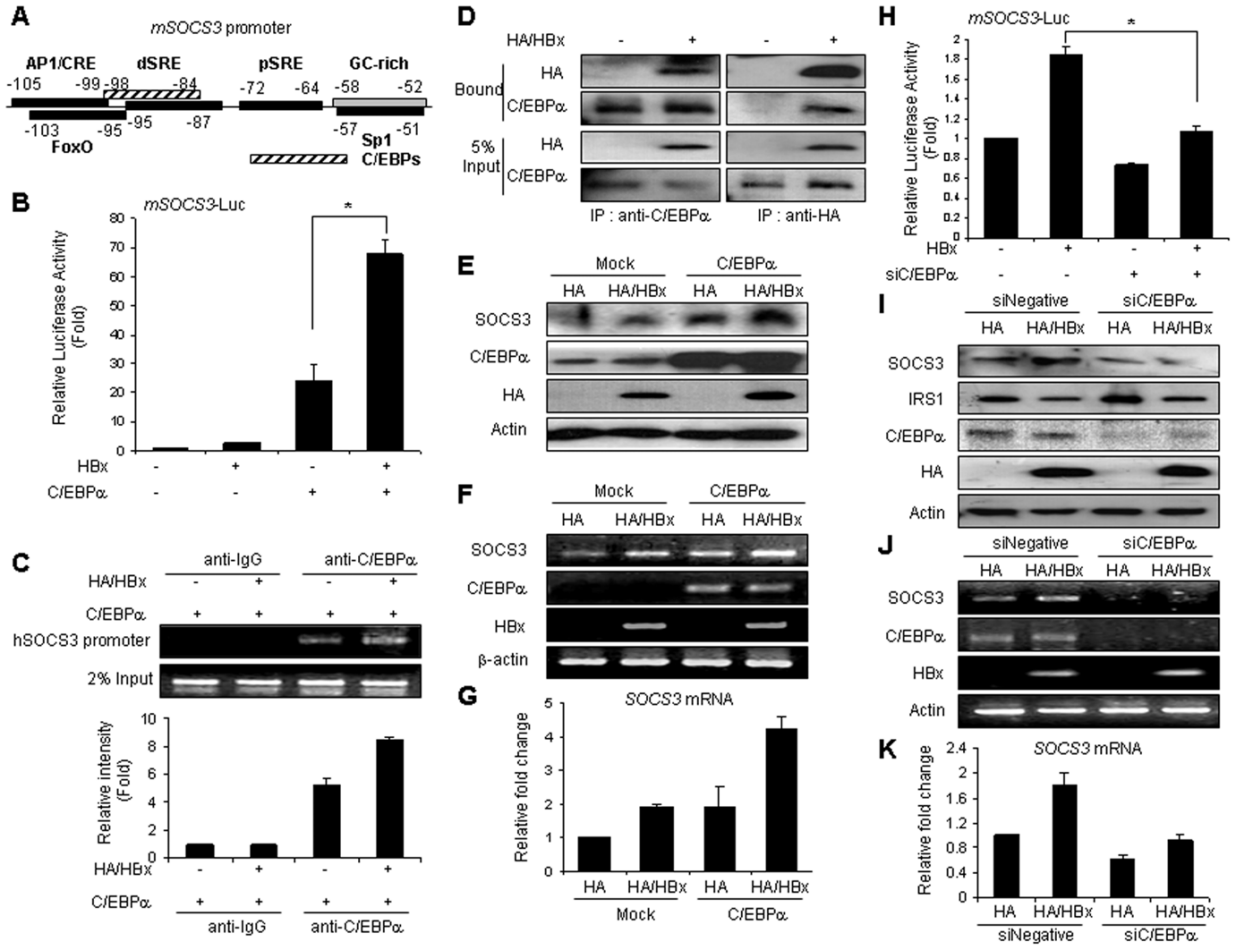
C/EBPα enhances HBx-induced SOCS3 expression. (A) Schematic representation of the transcriptional response elements on the murine SOCS3 promoter, showing a distal SRE (−95 to −87), a proximal SRE (−71 to −64), an AP1 element (−105 to −99), a FoxO motif (−103 to 95), a GC-rich region (−58 to −52), and a putative C/EBPs binding site (−98 to −84). (B) The effects of HBx and C/EBPα on SOCS3 promoter activity. HepG2 cells were transfected with SOCS3 reporter construct, HBx, and C/EBPα as indicated for 48 hr, after which the ratio of reporter activities was utilized to measure the promoter activity of SOCS3. *P<0.0001 compared with C/EBPα transfectants. (C) ChIP analysis on the SOCS3 promoter. HepG2 cells were transfected with expression vectors for HBx and C/EBPα as indicated and harvested for ChIP analysis with anti-C/EBPα antibody, or control IgG. The precipitated genomic fragments were amplified using primers flanking the C/EBPs binding sites on human SOCS3 promoters. Genomic DNA from total chromatin lysates was included as an input control. The precipitates were confirmed using quantitative real-time PCR. (D) The interaction between HBx and C/EBPα by coimmunoprecipitation. HA-tagged HBx was transiently expressed in HepG2 cells. Proteins in lysates were immunoprecipitated (IP) and immunoblotted (IB) with the indicated antibodies. (E) The induction of SOCS3 protein expression by HBx and C/EBPα. The C/EBPα protein was transiently expressed for 48 hr in Mock and stably HBx-transfected cells. Whole HepG2 cell extracts stably expressing HA-tagged HBx protein were immunoblotted with the SOCS3-specific antibody. (F), (G) The mRNA expression of SOCS3 by HBx and C/EBPα in RT-PCR (F) or quantitative real-time PCR (G). (H) The effect of C/EBPα knockdown on HBx-induced SOCS3 promoter activity. *P<0.0001 compared with the indicated control. (I) The expression of SOCS3 proteins by HBx in the C/EBPα knockdown system. Whole cell lysates were immunoblotted with indicated antibodies. (J). (K) The expression of SOCS3 mRNA by HBx in the C/EBPα knockdown system. Total RNA was extracted from the cells, and the levels of SOCS3 mRNA were determined via RT-PCR (J) and quantitative real-time PCR (K).

Based on the data for increasing the mRNA levels of SOCS3 by HBx, we further investigated the mechanisms for regulating SOCS3 expression by HBx. We conducted a functional analysis of the activation of the SOCS3 promoter. The structure of the SOCS3 gene has been previously determined [37]. The putative response elements present on the SOCS3 promoter are provided in Figure 4A. Using the ‘MOTIF’ database analysis program to identify any consensus sequences in the 5′-flanking region of this gene, we identified putative binding sites for several transcription factors, including C/EBPs. Compared to the mock-transfected cells, transfection with the HBx plasmid augmented the luciferase activity of the SOCS3 promoter over two folds, indicating that the induction of SOCS3 in response to HBx is mediated through the transcriptional activation of the SOCS3 gene. In order to investigate the possible involvement of transcription factors or coactivators with HBx on this promoter, we cotransfected HBx and transcription factors/coactivators, such as SREBP-1a, SREBP-1c, C/EBPα, CBP, p300, and PGC-1α, into HepG2 cells. In particular, C/EBPα increased both the basal and HBx-cotransfected luciferase expression of this promoter (Figure 4B). Other transcriptional regulators did not induce the HBx-mediated transactivation of the SOCS3 promoter (data not shown). ChIP assays were conducted in order to further evaluate the binding of C/EBPα to this promoter. C/EBPα bound on this promoter and HBx expression increased its binding to the promoter (Figure 4C).

In order to assess the potential *in vivo* interaction between endogenous C/EBPα and HBx proteins involved with SOCS3 expression, HepG2 cells were transfected with HA-tagged HBx gene, and anti-C/EBPα immunoprecipitation was conducted, followed by immunoblotting with anti-HA antibody. As shown in Figure 4D, HBx proteins interacted with C/EBPα via anti-HA antibody immunoprecipitation (*left*). And the interaction with HBx and C/EBPα was also refined using anti-HA immunoprecipitation, followed by Western blotting with antibody against C/EBPα (*right*). These results suggest that the functional synergism of HBx and C/EBPα by interaction of both proteins can induce the SOCS3 expression. Transfection of C/EBPα into HBx-expressed stable cells induced a marked increase in the protein and the mRNA levels of SOCS3 (Figure 4E, F). The mRNA induction in quantitative real-time PCR was confirmed for SOCS3 expression (Figure 4G).

In an effort to examine whether HBx performs a function in SOCS3 gene expression by C/EBPα, we attempted to knockdown of C/EBPα using specific siRNA for C/EBPα. As shown in Figure 4H, siC/EBPα-transfected cells did not induce SOCS3 promoter activity, even in the transfection cells of HBx. Also, the protein levels of SOCS3 were confirmed using Western blotting (Figure 4I). The siC/EBPα-transfected cells did not augment the protein expression of SOCS3 and the expression levels of IRS1 were increased noteworthily. As shown in Figure 4J and 4K, the mRNA levels of SOCS3 in the RT-PCR and quantitative real-time PCR was also not increased by HBx in the siC/EBPα-transfected cells. These results showed that the association of C/EBPα and HBx is required for the induction of SOCS3 expression via the efficient recruitment of this promoter.

### HBx-activated STAT3 is involved in SOCS3 expression

**Figure 5:**
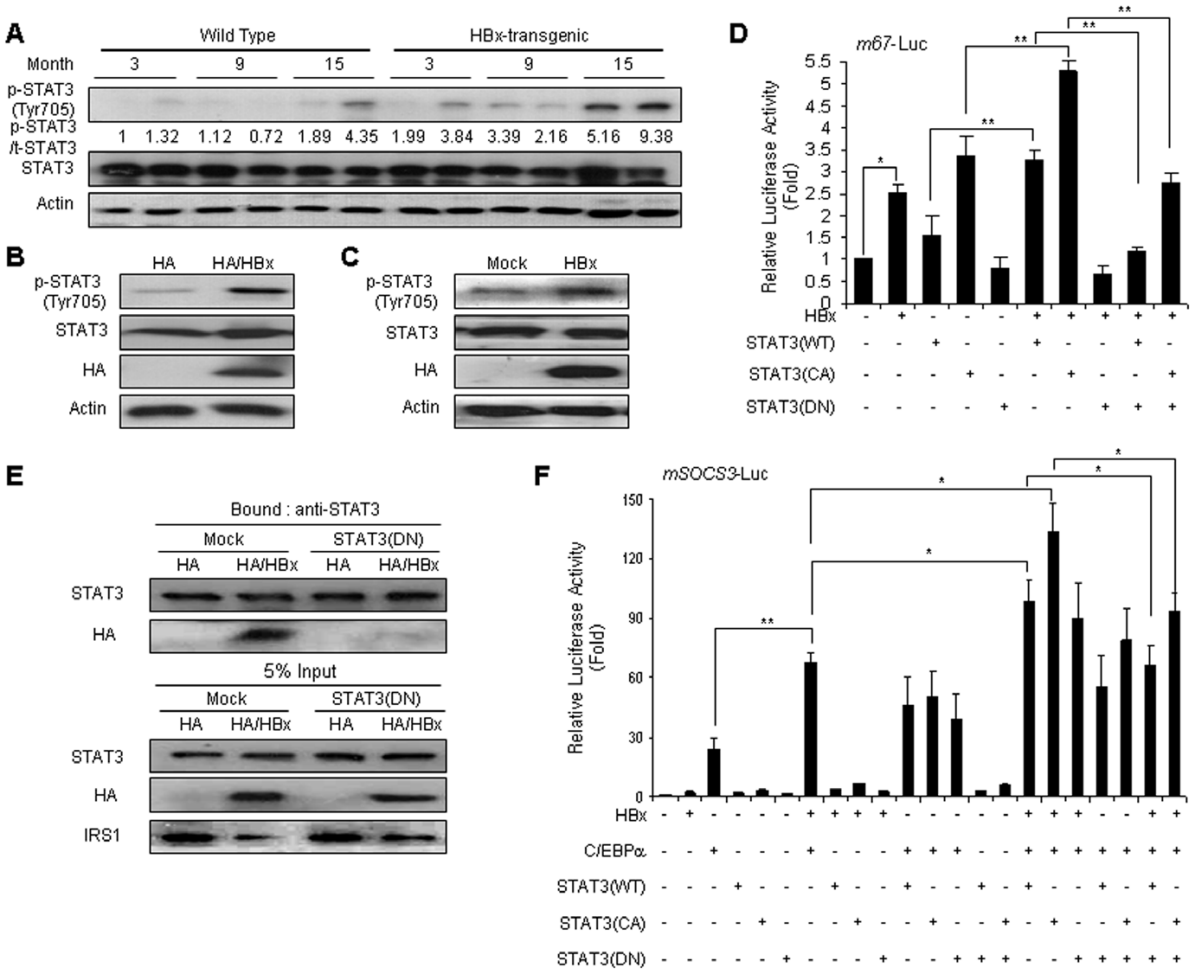
STAT3 activation is associated with HBx-induced SOCS3 expression. (A) The activation of STAT3 via HBx-induced tyrosine phosphorylation. Proteins in liver extracts from control and HBx-transgenic mice were immunoblotted with anti-STAT3 and anti-phospho-STAT3 (Tyr705) antibodies. (B), (C) The effect of HBx protein on STAT3 phosphorylation in HepG2 cells. HepG2 cell extracts stably (B) and transiently (C) transfected with HBx were immunoblotted with the STAT3 and phosphor-STAT3 (Tyr705) specific antibodies. (D) Transactivation of STAT3 by HBx expression. The m67-Luc (150 ng) was cotransfected into HepG2 cell with the eukaryotic expression vectors for the wild type (WT), constitutive active (CA), or dominant negative (DN) construct for STAT3 with or without HBx expression vectors. The luciferase activity was measured and the values are expressed as the means ± s.d. for at least four independent experiments. (E) The activation of STAT3 via interaction with HBx proteins. HA-tagged HBx and dominant negative STAT3 constructs were transfected into HepG2 cells. Proteins in lysates were immunoprecipitated with anti-STAT3 and immunoblotted with anti-STAT3 and anti-HA. (F) Luciferase activity of SOCS3 promoter dependent on the expression of HBx, C/EBPα, and STAT3 (WT, CA, and/or DN). The values were expressed as the means ± SD. *P<0.05 and **P<0.0001 compared with the indicated controls.

The tyrosine phosphorylation and activation of STATs results in the upregulation of STAT target genes, including three families of inhibitory proteins, the protein inhibitors of activated STATs (PIAS), the SH2-containing phosphatase (SHP), and SOCSs [38], [39]. Also, previous reports have shown that HBx is capable of activating STAT3 via some mechanisms [40], [41]. We attempted to determine whether HBx proteins were responsible for the activation of STAT3 in our HBx-transgenic mice and cell lines, given by the phosphorylation at Tyr705 [42], [43]. The highest levels of Tyr705 phosphorylation of STAT3 were detected in 15-month-old HBx-transgenic mice (Figure 5A) and forced HBx expression also increased STAT3 phosphorylation in the stable and transient transfectants (Figures 5B and 5C). To further substantiate our results, we examined whether HBx proteins promote activation of STAT3 transcription factor. Transient transfection of the m67-Luc construct, a reporter gene with STAT3 DNA binding sites [30], into HepG2 cells with or without HBx was used to test transcriptional activation by HBx-induced STAT3 phosphorylation. Upon induction of tyrosine phosphorylation of STAT3 by HBx expression, transcriptional stimulation by wild type (WT)-STAT3 became evident and a further increase transcription was also achieved by constitutive active (CA)-STAT3 (STAT3-C, which is dimerized by cysteine-cysteine residues instead of pY-SH2 interactions [30]) constructs). However, dominant negative (DN, Tyr705 mutated with Phe) STAT3 significantly decreased the HBx-induced STAT3 (WT or CA) activation (Figure 5D).

The immunoprecipitation of STAT3 and subsequent Western blot analysis showed that the STAT3 interacted with HBx. Consistent with the enhanced phosphorylation of STAT3 in the presence of HBx proteins, DN-STAT3 markedly disrupted the interaction of these proteins (Figure 5E). This result suggests that the activation of STAT3 by interaction with HBx proteins can induced the SOCS3 expression. Also, we subsequently detected the correlation between C/EBPα and STAT3 with regard to the expression of SOCS3. As shown in Figure 5F, the promoter activities of SOCS3 were higher in the cotransfection of HBx, C/EBPα, and STAT3 (WT and CA). The cotransfection of DN-STAT3 impaired the activation of the SOCS3 promoter. These results consistently indicate that HBx-induced C/EBPα and STAT3 activation may be an upstream regulator of SOCS3 expression.

### HBx attenuates hepatic insulin signaling

**Figure 6:**
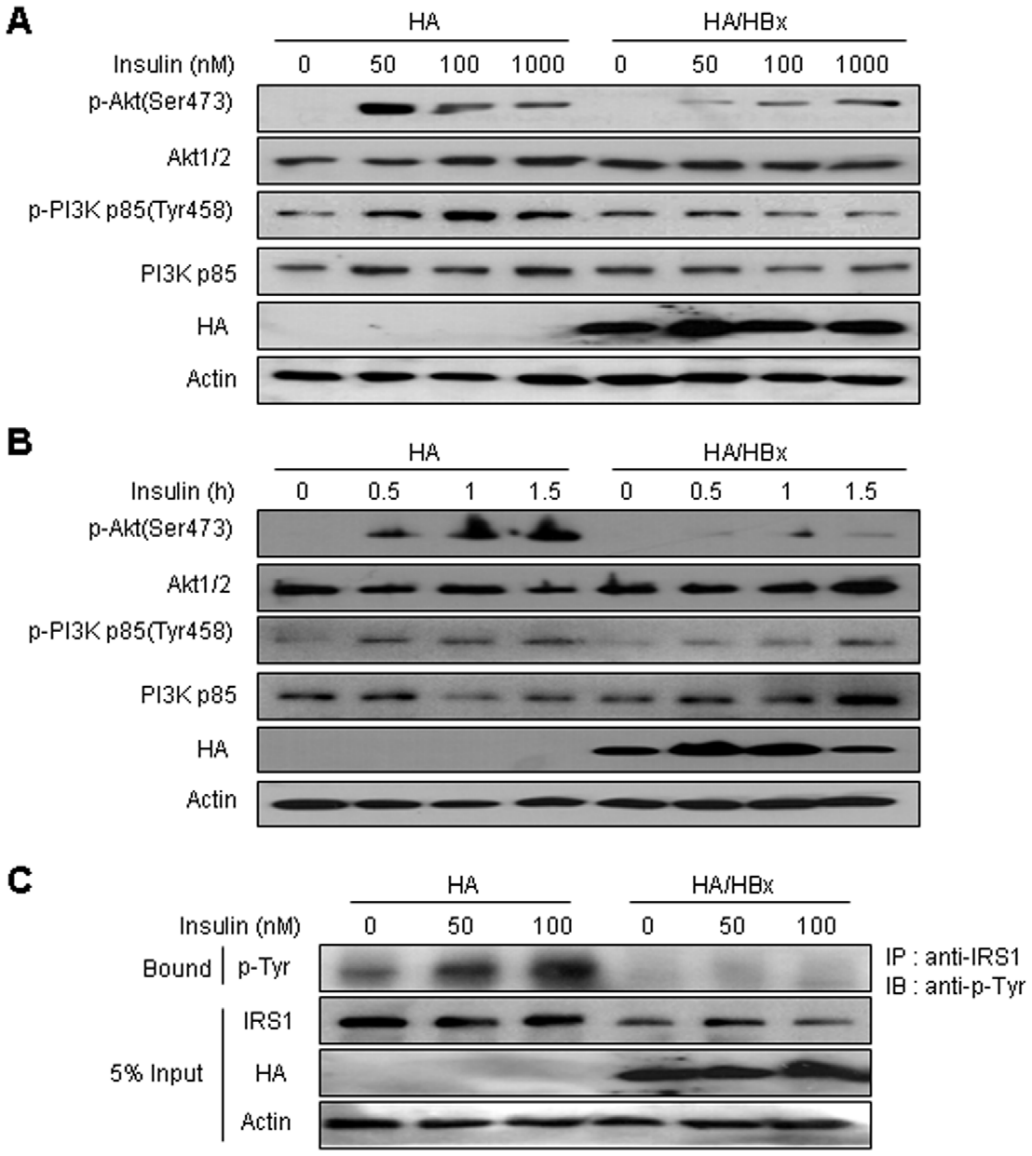
HBx disturbs insulin signaling in hepatic cells. (A) The effect of HBx on the insulin-induced phosphorylation of the p85 subunit of PI3K and Akt in the dose-response manner. Control and HBx-expressed stable cells were incubated in the presence of insulin for 60 minutes. Whole cell lysates were subjected to immunoblotting for phosphor-p85 (Tyr458) and phosphor-Akt (Ser473). (B) The effect of HBs on the insulin-treated phosphorylation of p85 and Akt in the time-course manner. Control and HBx stable cells were treated in the presence of 100 nM insulin for indicated times. Total cell lysates were examined to Western blotting for phosphor-p85 (Tyr458) and phosphor-Akt (Ser473). (C) The effect of HBx on the insulin-stimulated tyrosine phosphorylation of IRS1. Mock and HBx-expressed stable cells were treated for 60 minutes in the presence of 100 nM insulin. From the total cell extracts, IRS1 proteins were immunoprecipitated (IP), corresponding with the anti-IRS1 antibody, followed by immunoblotting with anti-phosphotyrosine antibody.

In order to confirm that HBx-induced IRS1 downregulation resulted in the impairment of insulin signaling, HBx-constitutive expressing HepG2 cells were treated with insulin in concentrations from 0 to 1000 nM for 1 hour, and the phosphorylation of p85 subunit of PI3K (Tyr458) and Akt (Ser473) were evaluated. The insulin-induced phosphorylation of the p85 subunit of PI3K and Akt were detected in HepG2 cells transfected with empty vector, even though the concentration of insulin was lower. However, lower levels of the phosphorylation of these proteins were detected in the HBx-expressing cells (Figure 6A). Total levels of PI3K-p85 and Akt remained unchanged by insulin treatment. We also observed that HBx-transfected cells were induced a significant decrease in the phosphorylation of p85 and Akt in response to insulin treatment in the time-dependent manner, compared to mock-transfeced cells (Figure 6B).

Furthermore, we assessed the tyrosine phosphorylation levels of IRS1 immunoprecipitates in the stable transfectants of HBx genes. Mostly, insulin-induced tyrosine phosphorylation of IRS1 can positively regulate the insulin signaling, in opposition to phosphorylation on Ser307 (Figure 1A) [44]. As expected, the levels of IRS1 tyrosine phosphorylation were reduced in the HBx-expressing cells treated with insulin, as compared to the control vector-transfected cells (Figure 6C).

### HBx disrupts insulin-induced PEPCK and G6Pase downregulation

**Figure 7:**
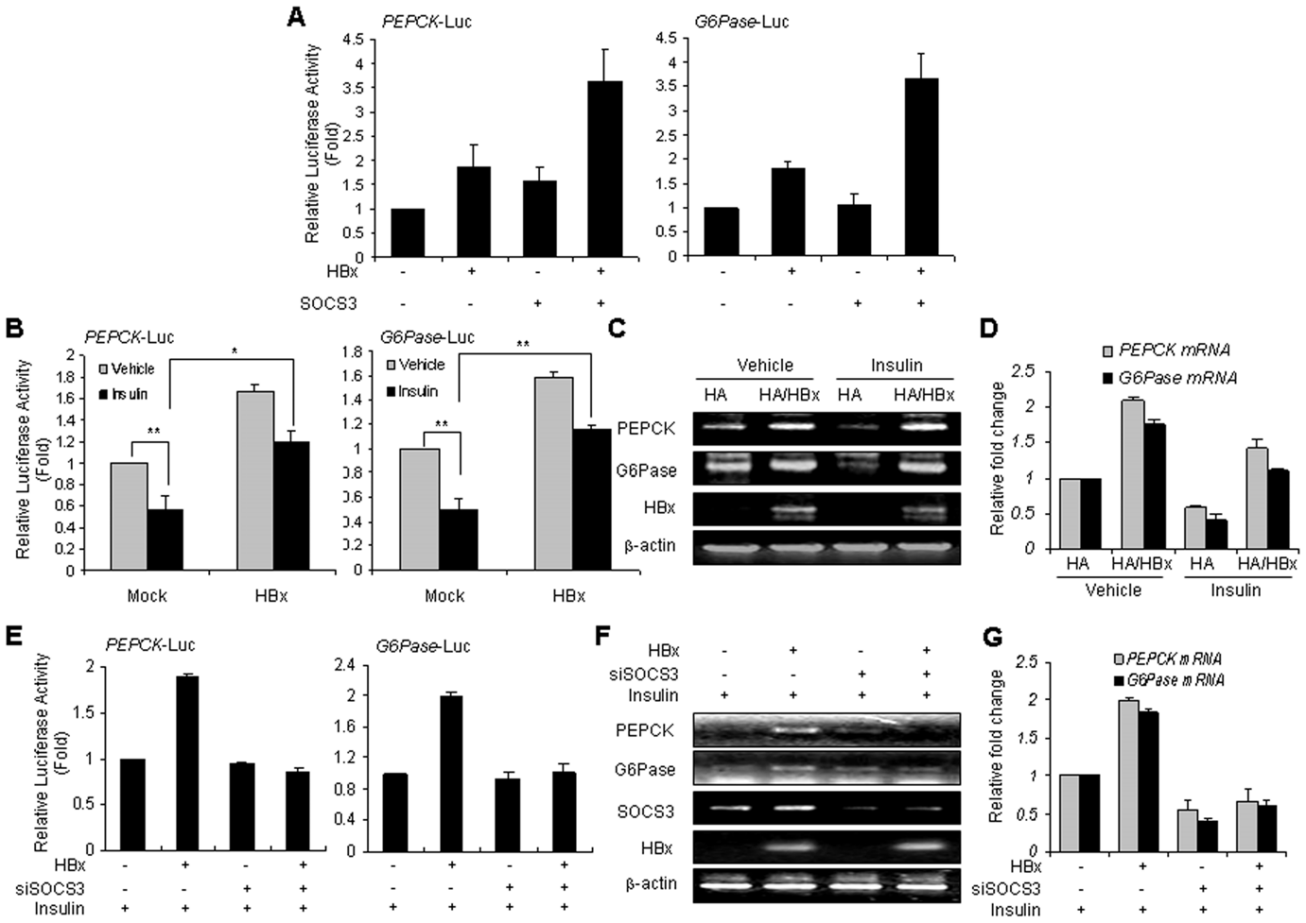
HBx suppresses insulin-inhibitory expression of gluconeogenic genes. (A) The promoter activity of PEPCK and G6Pase by HBx and SOCS3. HepG2 cells were transfected with two promoters of PEPCK (*left*) and G6Pase (*right*) and expression vectors of HBx, and SOCS3. (B) The inhibition of insulin-mediated promoter activity of PEPCK and G6Pase by HBx proteins. HepG2 cells were transiently transfected with PEPCK (*left*) or G6Pase (*right*) promoter construct and HBx in the presence or absence of insulin (100 nM). Luciferase assay was conducted to assess the promoter activity of PEPCK and G6Pase, and the error bars represent the standard deviation. *P<0.05 and **P<0.005 compared with indicated controls. (C), (D) Control and HBx stable cells were incubated for 1 h in the presence of insulin (100 nM). Total RNA was also isolated and subjected to RT-PCR analysis (C) or quantitative real-time PCR (D) with primers for PEPCK and G6Pase. β-Actin or GAPDH expression served as a control. (E) The promoter activity of PEPCK and G6Pase by HBx and RNAi against SOCS3 in the presence of insulin. HepG2 cells were transiently transfected with PEPCK (*left*) or G6Pase (*right*) promoter construct and HBx and/or RNAi for SOCS3 in the presence of insulin (100 nM). Luciferase assay was conducted to assess the promoter activity of PEPCK and G6Pase. (F), (G) The mRNA expression of PEPCK and G6Pase by HBx and/or siSOCS3 in treatments of insulin. Total RNA was isolated and subjected to RT-PCR (F) or quantitative real-time PCR (G).

Insulin reduces gluconeogenesis via the specific transcriptional inhibition of PEPCK and G6Pase [45]. Thus, we attempted to determine whether the attenuation of insulin signaling by HBx disrupts the actions of insulin with regard to the suppression of PEPCK and G6Pase expression. It was noted that the overexpression of HBx and SOCS3 proteins enhanced the promoter activity of PEPCK and G6Pase (Figure 7A). Furthermore, as shown in Figure 7B, although insulin attenuated the expression of the PEPCK and G6Pase genes, HBx inhibited the insulin-induced suppression of the promoter activity of these genes. We also verified the mRNA levels of these genes in the RT-PCR and quantitative real-time PCR (Figure 7C and 7D). Additionally, we observed that RNAi against SOCS3 could not induce PEPCK and G6Pase promoter activity in the presence of HBx (Figure 7E) and confirmed the mRNA levels of PEPCK and G6Pase in the RT-PCR (Figure 7F) and quantitative real-time PCR (Figure 7G). Collectively, these results demonstrate that HBx induces decreased insulin signaling, resulting in the inhibition of gluconeogenesis in hepatocytes.

## Discussion

As it has been estimated that approximately 53% of HCC cases worldwide are associated with HBV, research into HBV infection has been focused principally on the pathogenesis of HCC. However, it is possible that the deregulation of a number of metabolic components in HBV-infected livers may contribute to the pathogenesis of advanced liver diseases. Among the four proteins that originate from the HBV genome, such as polymerase, surface, core, and HBx proteins, HBx is reported to be associated with HBV-related pathogenesis [46]. Previous reports have demonstrated that HBx proteins induce fatty liver diseases via regulating of the expression of lipid synthesis-related genes in transgenic mice and hepatic cells [5], [6], and hepatic inflammation is observed frequently in HBx-transgenic mice [6]. Some reports are demonstrated that patients with HBV infection show an association between steatosis and insulin resistance, with its clinical concomitants of obesity, hyperglycemia, and hypertriglyceridemia [47].

HBV should be considered to exert synergistic effects with chronic inflammation [48]. Proinflammatory proteins, including TNF-α, IL-6, and IL-1β, appear to participate in the induction and maintenance of the subacute inflammatory state associated with the accumulation of fat. We also noted that the mRNA levels of TNF-α, IL-6, IL-1β, cyclooxygenase-2 (COX-2), C-reactive protein (CRP), and matrix metalloproteinase-9 (MMP-9) expression were increased in the presence of HBx (data not shown). Presumably, HBx-mediated fat accumulation may be associated with the increased production of these cytokines in response to viral hepatic infections or inflammation. Therefore, it could need to identify the relationship with chronic HBV infection, inflammation, and inflammation-related liver dysfunction, such as disruption of insulin signaling. Collectively, based on the fact that HBV, especially HBx proteins, induces hepatic steatosis and inflammation, we examined the possible association with HBx and insulin signaling.

The SOCS proteins are the most thoroughly studied inhibitors of Jak/STAT signaling pathway and include 8 members (cytokine inducible SH2 domain protein - CIS and SOCS 1 to 7), which are expressed at low levels in unstimulated cells. After cell activation, their expression increases, thereby inhibiting activated STATs [13], [26], [49]. In this study, we showed that HBx-induced C/EBPα and STAT3 activation was accompanied resulting in SOCS3 expressions were elevated. Also, given our observations that HBx activates the STAT3-SOCS3 regulatory pathway via cooperation with C/EBPα, the activation of this pathway is resulted in HBx-induced IRS1 degradation. As IRS proteins constitute a critical link in hepatic insulin signaling, the reduced expression of IRS proteins in the liver may result in inhibition of insulin signaling [50].

Compared with HCV infection, the association between HBV infection, insulin signaling, and its complications have been less clearly identified. The recent report was demonstrated that in patients with HBV infection, the relationship between host characteristics such as obesity and type 2 diabetes and HBV infection was determined [47]. It was reported that obesity and diabetes are predictors of HCC risk, possible depending on HBV infection status [51]. In this study, we suggest the molecular mechanisms resulting in the suppression of insulin signaling by HBx proteins. However, future research may be required to demonstrate the physiological significance of the disruption of insulin signaling by HBV infection, especially HBx expression, and host and/or external factors. Additionally, it is possible that HBx-induced cellular stress, such as mitochondrial dysfunction, oxidative stress, and endoplasmic reticulum stress, can be associated with inhibition of insulin signaling. Studies might also be necessary to explain a number of mechanisms, which can be associated with liver dysfunctions.

In conclusion, our data indicate that HBx proteins are able to augment SOCS3 expression and decrease IRS1 proteins, thereby causing impairments of hepatic insulin signaling and inhibition of hepatic insulin action. The results of the present study provide new insight into the pathogenesis of HBV infection underlying the deregulation of components of the insulin signaling framework and chronic hepatic metabolic changes.
